# You don’t have to tell a story! A registered report testing the effectiveness of narrative versus non-narrative misinformation corrections

**DOI:** 10.1186/s41235-020-00266-x

**Published:** 2020-12-09

**Authors:** Ullrich K. H. Ecker, Lucy H. Butler, Anne Hamby

**Affiliations:** 1 School of Psychological Science (M304), University of Western Australia, 35 Stirling Hwy, Perth, 6009 Australia; 2 College of Business and Economics, Boise State University, 1910 University Drive, Boise, ID 83725 USA

**Keywords:** Misinformation, Continued influence effect, Myth debunking, Narrative processing, Stories

## Abstract

Misinformation often has an ongoing effect on people’s memory and inferential reasoning even after clear corrections are provided; this is known as the continued influence effect. In pursuit of more effective corrections, one factor that has not yet been investigated systematically is the narrative versus non-narrative format of the correction. Some scholars have suggested that a narrative format facilitates comprehension and retention of complex information and may serve to overcome resistance to worldview-dissonant corrections. It is, therefore, a possibility that misinformation corrections are more effective if they are presented in a narrative format versus a non-narrative format. The present study tests this possibility. We designed corrections that are either narrative or non-narrative, while minimizing differences in informativeness. We compared narrative and non-narrative corrections in three preregistered experiments (total *N* = 2279). Experiment 1 targeted misinformation contained in fictional event reports; Experiment 2 used false claims commonly encountered in the real world; Experiment 3 used real-world false claims that are controversial, in order to test the notion that a narrative format may facilitate corrective updating primarily when it serves to reduce resistance to correction. In all experiments, we also manipulated test delay (immediate vs. 2 days), as any potential benefit of the narrative format may only arise in the short term (if the story format aids primarily with initial comprehension and updating of the relevant mental model) or after a delay (if the story format aids primarily with later correction retrieval). In all three experiments, it was found that narrative corrections are no more effective than non-narrative corrections. Therefore, while stories and anecdotes can be powerful, there is no fundamental benefit of using a narrative format when debunking misinformation.

## Significance statement

Misinformation often has an ongoing effect on people’s reasoning even after they receive corrections. Therefore, to reduce the impact of misinformation, it is important to design corrections that are as effective as possible. One suggestion often made by front-line communicators is to use stories to convey complex information. The rationale is that humans are uniquely “tuned” to stories, such that the narrative format facilitates understanding and retention of complex information. Some scholars have also suggested that a story format may help overcome resistance to corrections that threaten a worldview-consistent misconception. It is, therefore, a possibility that misinformation corrections are more effective if they are presented in a narrative versus a non-narrative, more fact-oriented format. The present study tests this possibility. We designed narrative and non-narrative corrections that differ in format while conveying the same relevant information. In Experiment 1, corrections targeted misinformation contained in fictional event reports. In Experiment 2, the corrections targeted false claims commonly encountered in the real world. Experiment 3 used real-world claims that are controversial, in order to test the notion that a narrative format may facilitate corrective updating primarily when it serves to reduce resistance to correction. In all experiments, we also manipulated test delay, as any benefit of the narrative format may only arise in the short term (if the story format aids primarily with initial understanding) or after a delay (if the story format aids primarily with later memory for the correction). It was found that narrative corrections are no more effective than non-narrative corrections. Therefore, while stories and anecdotes can be powerful, there is no fundamental benefit of using a narrative format when debunking misinformation. Front-line communicators are advised to focus primarily on correction content—while there will be cases where a narrative frame will naturally lend itself to a particular debunking situation, this study suggests that a narrative approach to debunking will not generally be superior.

## Introduction

The contemporary media landscape is awash with false information (Lazer et al. 2018; Southwell and Thorson 2015; Vargo et al. 2018). Misinformation featured in the media ranges from preliminary accounts of newsworthy events that are superseded by more accurate accounts as evidence accrues (e.g., a wildfire is initially believed to be arson-related but is later found to have been caused by a fallen power pole), to commonly encountered “myths” about causal relations (e.g., alleged links between childhood vaccinations and various negative health outcomes), to strategically disseminated disinformation that intends to deceive, confuse, and sow social division (e.g., doctored stories intended to discredit or denigrate a political opponent during an election campaign; see Lewandowsky et al. 2017).

From a psychological perspective, an insidious aspect of misinformation is that it often continues to influence people’s reasoning after a clear correction has been provided, even when there are no motivational reasons to dismiss the correction; this is known as the continued influence effect (CIE; Johnson and Seifert 1994; Rapp and Salovich 2018; Rich and Zaragoza 2016; Thorson 2016; for reviews see Chan et al. 2017; Lewandowsky et al. 2012; Walter and Tukachinsky 2020). Theoretically, the CIE is thought to arise either from failure to integrate the corrective information into the mental model of the respective event or causal relationship or from selective retrieval of the misinformation (e.g., familiarity-driven retrieval of the misinformation accompanied by failure to recollect the correction; see Ecker et al. 2010; Gordon et al. 2017, 2019; Rich and Zaragoza 2016; Walter and Tukachinsky 2020).

Given the omnipresence of misinformation, it is of great importance to investigate the factors that make corrections more effective. For example, corrections are more effective if they come from a more credible source (Ecker and Antonio 2020; Guillory and Geraci 2013; Vraga et al. 2020), contain greater detail (Chan et al. 2017; Swire et al. 2017), or a greater number of counterarguments (Ecker et al. 2019). However, even optimized debunking messages typically cannot eliminate the continued influence of misinformation, not even if reasoning is tested immediately after a correction is provided, let alone after a delay (see Ecker et al. 2010, 2020a; Paynter et al. 2019; Rich and Zaragoza 2016; Swire et al. 2017; Walter and Tukachinsky 2020). Thus, additional factors to enhance the effectiveness of corrections need to be identified. The present paper is thus concerned with one particular avenue that might make corrections more effective, which is important because greater correction effects mean smaller continued influence effects.

Specifically, one piece of advice often given by educators and science communicators regarding the communication of complex information, such as misinformation corrections, is to use stories (e.g., Brewer et al. 2017; Caulfield et al. 2019; Dahlstrom 2014; Klassen 2010; Marsh et al. 2012; Shelby and Ernst 2013). For example, Shelby and Ernst (2013) argued that part of the reason why some misconceptions are common among the public is that disinformants use the power of storytelling, while fact-checkers often rely exclusively on facts and evidence. Indeed, people seem to be influenced by anecdotes and stories more so than stated facts or statistical evidence in their medical decision-making (Bakker et al. 2019; Fagerlin et al. 2005), risk perceptions (Betsch et al. 2013; de Wit et al. 2008; Haase et al. 2015), behavioral intentions and choices (Borgida and Nisbett 1977; Dillard et al. 2018), and attitudes (Lee and Leets 2002).

Despite some fragmentation in defining what constitutes a story, researchers generally agree that stories are defined by their chronology and causality: they depict characters pursuing goals over time, and may feature access to characters’ thoughts and emotions (Brewer and Lichtenstein 1982; Bruner 1986; Pennington and Hastie 1988; Shen et al. 2014; van Krieken and Sanders 2019). Research on narrative processing often contrasts narrative messages with non-narrative formats (such as those that feature statistics or facts, descriptive passages, or texts that use a list-based, informative format; sometimes these are also called “expository” or “informational” texts; Ratcliff and Sun 2020; Reinhart 2006; Shen et al. 2014; Zebregs et al. 2015b). Though non-narrative formats may differ in form and substance, they often share an abstract, logic-based, decontextualized message style (relative to narratives), and tend to evoke analytical processing. Research from advertising and consumer psychology suggests that even short messages featuring several lines of text can evoke narrative or analytical processing styles, based on their content (Chang 2009; Escalas 2007; Kim et al. 2017).

Stories can impact reasoning and decision making through several mechanisms (see Hamby et al. 2018; Shaffer et al. 2018). Compared to processing of non-narrative messages, narrative processing is usually associated with greater emotional involvement in the message (Busselle and Bilandzic 2008; Golke et al. 2019; Green and Brock 2000; Ratcliff and Sun 2020). While narrative and non-narrative messages can be cognitively engaging, the nature of engagement differs. Readers of narratives apply more imagery and visualization and may even report feelings of transportation into the world of the story, in which they experience story events as though they were happening to them personally (Bower and Morrow 1990; Green and Brock 2000; Hamby et al. 2018; Mar and Oatley 2008). Additionally, narrative processing tends to reduce resistance to message content; not only are narratives usually less overtly persuasive than their non-narrative counterparts, but audiences are often less motivated to generate counterarguments when processing narratives, as this would disrupt the enjoyable experience of immersion in the story (Green and Brock 2000; Krakow et al. 2018; Slater and Rouner 1996). Stories may thus lead to stronger encoding and comprehension of information embedded within because of the cognitive and emotional involvement they tend to evoke (Browning and Hohenstein 2015; Romero et al. 2005; Zabrucky and Moore 1999).

In addition, a story format may facilitate information retrieval (Bower and Clark 1969; Graesser et al. 1980). This may arise from the aforementioned enhanced processing at encoding, to the extent that enhanced encoding results in a more vivid and coherently integrated memory representation (Graesser and McNamara 2011). Bruner (1986) argued that the story format provides the most fundamental means by which people construct reality, and enhanced retrieval of information presented in story format may therefore also result from the fact that stories typically offer a structured series of retrieval cues (e.g., markers of spatiotemporal context or characters’ emotional states or introspections) that are consistent with the way in which people generally think. In the context of misinformation processing, a correction that is more easily retrieved during a subsequent reasoning task will naturally promote use of correct information and reduce reliance on the corrected misinformation (see Ecker et al. 2011).

However, the evidence regarding the persuasive superiority of the story format over non-narrative text is not entirely consistent. Some studies contrasting narrative and non-narrative formats of health-related messages found both formats equally able to effect changes to attitudes and behavioral intentions (Dunlop et al. 2010; Zebregs et al. 2015a). Greene and Brinn (2003) even reported that narratives were inferior to non-narrative texts in reducing use of tanning beds. Early meta-analyses found that narrative information is either less persuasive than statistical information (Allen and Preiss 1997) or that there is no clear difference in favor of either approach (Reinhart 2006). More recent meta-analyses, however, found stronger support for the narrative approach (e.g., Ratcliff and Sun 2020), while also highlighting that communication effectiveness depends on persuasion context: While Zebregs et al.’s (2015b) analysis found that narrative information was superior to statistical information when it comes to changing behavioral intentions, they found that statistical evidence had stronger effects on attitudes and beliefs. Shen, Sheer, and Li (2015) found that narratives were more effective than non-narrative communications when it came to fostering prevention but not cessation behaviors.

Similar to the approach taken in the present study, Golke et al. (2019) contrasted standard non-narrative texts with so-called informative narratives—enhanced fact-based texts that present essentially the same information as the standard non-narrative fact-based text, but in a storyline format. They found that the narrative format did not enhance reading comprehension and even reduced comprehension in two of their three experiments. Wolfe and Mienko (2007) found no retrieval benefit for informative narratives, and Wolfe and Woodwyk (2010) reported that readers showed enhanced integration of new information with existing knowledge when reading non-narrative texts compared to informative narratives. In the context of misinformation corrections, this may suggest that narrative elements may distract the reader from the core correction and/or that non-narrative corrections may facilitate integration of the correction into the reader’s mental model, which may render them more effective than informative-narrative corrections (see Kendeou et al. 2014).

In sum, while there may be some rationale in using a story format to correct misinformation, the question of whether corrections are more effective when they are given in a story format rather than a non-narrative format remains to be empirically tested. To the best of our knowledge, only one study has investigated the effectiveness of narrative corrections. Sangalang et al. (2019) explored whether narrative corrections could reduce smokers’ misinformed beliefs about tobacco. Results were inconclusive, as a narrative correction was found to reduce misconceptions in only one of the two experiments reported. Importantly, this study did not contrast narrative and non-narrative corrections. This was the aim of the present study.

In three experiments, we contrasted corrections that focus on factual evidence with corrections designed to present the same amount of relevant corrective information, but in a narrative format. In designing these corrections, we took inspiration from the broader literature on narrative persuasion reviewed above (in particular, Shen et al. 2014; van Krieken and Sanders 2019) to ensure narrative and non-narrative corrections differed on relevant dimensions. Narrative corrections featured characters’ experiences and points of view, quotes, chronological structure, and/or some form of complication or climax, whereas non-narrative corrections focused more on the specific facts and pieces of evidence, had a less engaging and emotive writing style, and adhered more closely to an inverted-pyramid format (essential facts followed by supportive evidence and more general background information).

In order to investigate the robustness of potential narrative effects, we aimed to correct both fictional event misinformation and real-world misconceptions: Experiment 1 used fictional event reports of the type used in most research on the continued influence effect (e.g., Ecker et al. 2017). The reports first introduced a piece of critical information that related to the cause of the event, while the correction refuted that piece of critical information. Participants’ inferential reasoning regarding the event, in particular their reliance on the critical information, was then measured via questionnaire. Experiment 2 corrected some common real-world “myths” while affirming some obscure facts (as in Swire et al. 2017). We measured change in participants’ beliefs, as well as their posttreatment inferential reasoning relating to the false claims. Experiment 3 examined the effect of correction format in the context of more controversial, real-world claims. To the extent that a narrative advantage arises from reduced resistance to the corrective message (see Green and Brock 2000; Krakow et al. 2018; Slater and Rouner 1996), it should become particularly apparent with corrections of worldview-consistent misconceptions. We hypothesized that narrative corrections will generally be more effective at reducing misinformation-congruent reasoning and beliefs.

In all experiments, we additionally manipulated retention interval (i.e., study-test delay). The rationale for this is as follows: Any potential story benefit might arise immediately—to the extent that the narrative format boosts engagement with and comprehension of the correction, and thus facilitates its mental-model integration. However, a story benefit may only arise after a delay, to the extent that the narrative format facilitates correction retrieval at test, which will be more relevant after some delay-related forgetting has occurred. In other words, if the narrative format is beneficial for retrieval, this benefit may not become apparent in an immediate test because participants are likely to remember both the narrative and the non-narrative correction just minutes after encoding; however, a story benefit may emerge with a delay, when the corrections are no longer “fresh” in one’s memory (see Ecker et al. 2020a; Swire et al. 2017).

## Experiment 1

### Method

Experiment 1 presented fictional event reports in four conditions. There were two control conditions: One featured no misinformation (noMI condition), another featured a piece of misinformation that was not corrected (noC condition). The two experimental conditions corrected the initially-provided misinformation using either a non-narrative (NN) or narrative (N) correction. The test phase followed the study phase either immediately or after a 2-day delay. The experiment thus used a mixed within-between design, with the within-subjects factor of condition (noMI; NN; N; noC), and the between-subjects factor of test delay (immediate; delayed).

#### Participants

Participants were US-based adults recruited via the platform Prolific.^1^ An a priori power analysis (using G*Power 3; Faul et al. 2007) suggested a minimum sample size of *N* = 352 to detect a small difference between the two within-subjects experimental conditions (i.e., NN vs. N; effect size *f* = 0.15; *α* = 0.05, 1 − *β* = 0.8). As the core planned analyses tested for effects in each delay condition separately, and to achieve an adequate sample size post-exclusions, it was thus decided to aim for a total of *N* = 800 participants pre-exclusions (*n* = 400 per delay condition). Due to inevitable dropout in the delayed condition (estimated at 20%), this condition was oversampled by a factor of 1.25 (i.e., 500 participants completed the study phase).

A total of 844 participants completed Experiment 1. Retention of participants in the delayed condition was slightly greater than expected (approx. 89%). After applying preregistered exclusions (described in “Results” section), the final sample size for analysis was *N* = 770 (*n* = 357 and *n* = 413 in the immediate and delayed conditions, respectively); the sample comprised 383 men, 379 women, and 8 participants of undisclosed gender; mean age was *M* = 34.01 years (SD = 11.56, age range 18–89).

#### Materials

Experiment 1 used four fictitious event reports detailing four different newsworthy events (e.g., a wildfire); each report comprised two articles. In the study phase, participants were presented with all four reports in the four different conditions. In three of the conditions, the report’s first article contained a piece of misinformation (e.g., the wildfire was caused by arson; this was simply omitted from the report in the no-misinformation condition); in these conditions, the report’s second article either contained or did not contain a correction. If a correction was provided, it was given in either a non-narrative format (e.g., explaining that an investigation had found that a rotten power pole had fallen and the power line had melted on the ground, starting the fire) or a narrative format (e.g., explaining that a fire chief inspected the scene, found the power pole, noticed the rot, and discovered that the power line had melted on the ground, concluding it had started the fire). Narrative and non-narrative corrections thus presented the same critical corrective information, but differed in the way it was presented: Narrative corrections featured specific characters and a causally ordered description sequence; non-narrative corrections featured objective, generalized descriptions of the events (per our definition of narrative and non-narrative format; Brewer and Lichtenstein 1982; Bruner 1986; Pennington and Hastie 1988; Shen et al. 2014; van Krieken and Sanders 2019). All reports thus existed in four versions (matching the conditions; all report versions are provided in “Appendix”). We aimed to keep non-narrative and narrative reports as equivalent as possible in terms of informativeness, length, and reading difficulty. A pilot study confirmed that our narrative corrections were perceived as more “story-like” than the non-narrative corrections, and also as more vivid and more easily allowing the events to be imagined. By contrast, the two correction versions were rated as relatively comparable on informativeness and comprehensibility (for details, see “Appendix”). Assignment of event reports to experimental conditions, as well as condition and event order, was counterbalanced across participants using four different presentation sequences in a Latin-square design, as shown in Table 1.

**Table 1:** Presentation sequences (S1–4) used in experiment 1

	Pos 1	Pos 2	Pos 3	Pos 4
S1	A_noMI	B_NN	C_noC	D_N
S2	B_N	A_noC	D_NN	C_noMI
S3	C_NN	D_noMI	A_N	B_noC
S4	D_noC	C_N	B_noMI	A_NN

Sequences counterbalanced the assignment of event reports (A–D) to conditions (no-misinformation, noMI; non-narrative correction, NN; narrative correction, N; no correction, noC) as well as event and condition order across sequence positions (Pos 1–4). Assignment of presentation sequence to participants was randomized, with the constraint that a quarter of participants received each sequence

The test comprised a memory question and six inference questions per report. The memory questions were four-alternative-choice questions targeting an arbitrary detail provided twice in the report (once in each article; e.g., “The fire came close to the town of Cranbrook/Kimberley/Lumberton/Bull River”). The sole purpose of the memory questions was to ensure adequate encoding; data from participants who did not demonstrate adequate encoding were excluded from analysis (see exclusion criteria below). The inference questions were designed to measure misinformation-congruent inferential reasoning, following previous CIE research (e.g., Ecker et al. 2017). Five of the six inference questions per report were rating scales asking participants to rate their agreement with a misinformation-related statement on a 0–10 Likert scale (e.g., “Devastating wildfire intentionally lit” would be an appropriate headline for the report). One inference question was a four-alternative-choice question targeting the misinformation directly (e.g., “What do you think caused the wildfire? Arson/Lightning/Power line/None of the above”). Such measures have been found appropriate for online CIE studies (Connor Desai and Reimers 2019). All questions are provided in “Appendix”.

All materials were presented via experimental surveys designed and administered via Qualtrics (Qualtrics, Provo, UT). The survey file, including all materials, is available on the Open Science Framework (https://osf.io/gtm9z/). Surveys with immediate and delayed tests were necessarily run separately due to the need for different signup instructions (the immediate survey was run at the same time as the delayed test). Participants in the delayed condition were reminded via e-mail to complete the test phase 48 h after launch of the study phase; they had 48 h to complete from launch of the test phase but were encouraged to complete within 24 h.

The experiment took approximately 12 min. Participants in the immediate condition were reimbursed GBP1.50 (approx. US$1.95) via Prolific; participants in the delayed condition were reimbursed GBP0.70 (approx. US$0.90) for the study phase and GBP0.80 (approx. US$1.05) for the test phase.

#### Procedure

Initially, participants were provided with an ethics-approved information sheet. Participants were asked to provide an English proficiency rating (1: excellent to 5: poor), gender, and age information and indicate their country of residence. The four reports were then presented, with each article presented on a separate screen, with applied fixed minimum times (set at approx. 150 ms per word).

The test followed after a short (1-min, filled with a word puzzle) or long (2 days) retention interval. Participants were presented with a questionnaire for each report, each comprising the memory question and the six inference questions. The order of questionnaires followed the order of the reports in the study phase; the order of questions in each questionnaire was fixed (see “Appendix”).

Following the test phase, participants were given a “data use” question asking them to provide honest feedback on whether or not their data should be included in our analysis (“In your honest opinion should we use your data in our analysis? This is not related to how well you think you performed, but whether you put in a reasonable effort.”). This question could be answered with “Yes, I put in reasonable effort (1)”; “Maybe, I was a little distracted (2)”; or “No, I really wasn’t paying any attention (3)”.

### Results

Data analysis was preregistered at https://osf.io/svy6f; the data are available at https://osf.io/gtm9z/. Analysis adhered to the following procedure: First, exclusion criteria were applied. We excluded data from participants who (a) indicated they do not reside in the USA (*n* = 0); (b) indicated their English proficiency is only “fair” or “poor” (*n* = 3); (c) responded to the “data use” question with “No [do not use my data], I really wasn’t paying any attention” (*n* = 5); (d) failed three or more memory questions in the immediate test (*n* = 28), or all four in the delayed test (*n* = 15)^2^; (e) responded in a “cynical” manner by selecting the “none of the above” response option for all four multiple-choice inference questions (*n* = 1); and (f) responded uniformly (a response SD across all 20 raw rating-scale inference-question responses < 0.5; *n* = 22). Finally, to identify inconsistent, erratic responding, we calculated response SD for each set of five inference questions and then calculated mean SD across the four sets. We (g) excluded outliers on this measure, using the interquartile rule with a 2.2 multiplier (i.e., cutoff = Q3 + 2.2 × IQR; Hoaglin and Iglewicz 1987; *n* = 0).

**Fig. 1:**
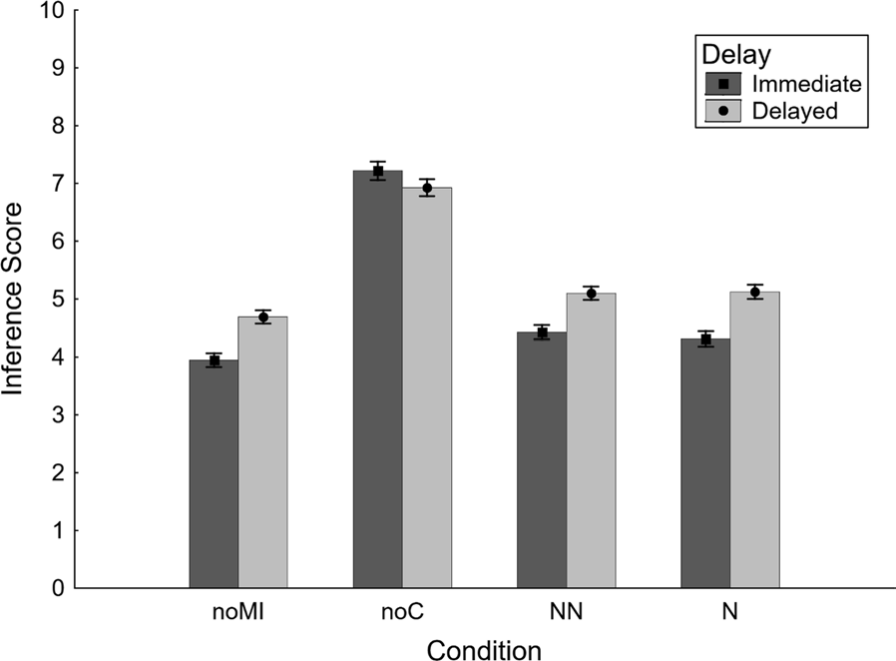
Mean inference scores across conditions in Experiment 1. noMI, no-misinformation; noC, no correction; NN, non-narrative; N, narrative. Greater values indicate greater misinformation reliance. Error bars indicate within-subjects standard error of the mean (Morey 2008)

We coded the multiple-choice inference-question responses as either 10 (misinformation option) or 0 (non-misinformation options). We then calculated four mean inference scores for the noC, NN, N, and noMI conditions; this was the main dependent variable, with greater scores reflecting greater misinformation reliance. We ran a two-way mixed ANOVA with factors condition (within-subjects) and delay (between-subjects) on inference scores (see Fig. 1). This yielded significant main effects of condition, *F*(3,2304) = 250.94, MSE = 4.79, *η*_p_^2^ = .246, *p* < .001, and delay, *F*(1,768) = 11.33, MSE = 15.77, *η*_p_^2^ = .015, *p* ≤ .001, which were qualified by a significant interaction, *F*(3,2304) = 10.75, *η*_p_^2^ = .014, *p* < .001, such that inference scores were higher after delay in all conditions but the no-correction condition. We tested the core hypothesis with planned contrasts, assessing the difference between NN and N conditions (planned contrast: NN > N; i.e., narrative correction more effective at reducing reliance on misinformation than non-narrative correction) in each delay condition; both contrasts were nonsignificant, *F*s < 1. There was thus no difference between non-narrative and narrative corrections.

We also tested the interaction contrast of NN versus N × immediate versus delayed. The direction of a potential interaction was not prespecified: We speculated that a potential narrative benefit may only emerge after a delay if the effect reflects retrieval facilitation, or may emerge immediately if it reflects stronger correction encoding or integration into the mental event model. However, the contrast was nonsignificant, *F* < 1.

To complement this frequentist analysis (and to quantify evidence in favor of the null), we ran Bayesian *t*-tests comparing NN and N in both delay conditions. In the immediate condition, this returned a Bayes Factor of BF_01_ = 12.26; in the delayed condition, we found BF_01_ = 17.76. This means that the data are approx. 12–18 times more likely under the null hypothesis of no difference between narrative conditions. This constitutes strong evidence in favor of the null (Wagenmakers et al. 2018).

Finally, for the sake of completeness, we ran an additional series of five secondary planned contrasts for each delay condition (see Table 2). Statistical significance was established using the Holm-Bonferroni correction, applied separately to each set of contrasts. These contrasts demonstrated that uncorrected misinformation increased reliance on the misinformation relative to the no-misinformation baseline and that corrections were very effective, strongly reducing misinformation reliance, albeit not quite down to baseline, which demonstrates the presence of a small continued influence effect.

**Table 2:** Secondary contrasts run in Experiment 1

#	Contrast	Effect	F(1,768)	η_p_^2^	p
	Immediate				
1	noMI < noC	Effect of uncorrected misinformation against no-misinformation baseline	360.89	.320	< .001*
2	noMI < NN	Continued influence effect of misinformation (non-narrative correction)	11.62	.015	≤ .001*
3	noMI < N	Continued influence effect of misinformation (narrative correction)	5.64	.007	.018*
4	noC > NN	Effectiveness of non-narrative correction relative to no-correction baseline	238.94	.237	< .001*
5	noC > N	Effectiveness of narrative correction relative to no-correction baseline	249.53	.245	< .001*
	Delayed				
1	noMI < noC	Effect of uncorrected misinformation against no-misinformation baseline	195.86	.203	< .001*
2	noMI < NN	Continued influence effect of misinformation (non-narrative correction)	9.85	.013	.002*
3	noMI < N	Continued influence effect of misinformation (narrative correction)	9.29	.012	.002*
4	noC > NN	Effectiveness of non-narrative correction relative to no-correction baseline	118.81	.134	< .001*
5	noC > N	Effectiveness of narrative correction relative to no-correction baseline	111.30	.127	< .001*

*indicates statistical significance following Holm-Bonferroni correction

We performed two additional analyses that were not preregistered. First, we tested whether correction effects were reduced after a delay, as would be expected based on previous research (e.g., Paynter et al. 2019; Swire et al. 2017). To this end, we tested the interaction contrast of immediate versus delayed test × no-correction versus (pooled) correction conditions. This yielded a significant result, *F*(1,768) = 20.49, MSE = 6.62, *η*_p_^2^ = .026, *p* < .001, confirming the expectation. Second, we tested for the effect of delay on memory performance, finding that as expected memory was better in the immediate test (*M* = .81; *SE* = .013) compared to the delayed test (*M* = .62, *SE* = .013), *F*(1,808) = 106.23, MSE = .07, *η*_p_^2^ = .116, *p* < .001 (this analysis included participants who failed exclusion criterion (d) related to memory performance).

### Discussion

Experiment 1 investigated whether corrections of event-related misinformation are more effective if presented in a narrative format. In line with much previous research (e.g., Chan et al. 2017; Walter and Tukachinsky 2020), we found a continued influence effect, in that corrected misinformation had a small but reliable effect on inferential reasoning. Also congruent with previous work, we found reduced memory and correction impact after a delay, which are both easily explained through standard forgetting of materials (see Paynter et al. 2019; Swire et al. 2017). However, results did not support the core hypothesis: narrative and non-narrative corrections were equally effective at reducing the effects of the misinformation. This suggests that the narrative format did not facilitate comprehension of the corrective information, its integration into the event model, nor its later retrieval during reasoning in a substantial manner. It is possible, however, that no narrative advantage was observed because the event reports provided sufficient narrative scaffolding in both conditions. In other words, to the extent that the events were already processed as narratives, it may have been easy to integrate the correction in either condition, and as such the format of the correction itself may have not provided additional benefit. It is, therefore, possible that a narrative advantage may only arise with misinformation that is not part of an event report. To test this, Experiment 2 used false real-world claims.

## Experiment 2

To examine the robustness and generality of the results of Experiment 1, Experiment 2 examined the effect of narrative versus non-narrative corrections on real-world beliefs.

### Method

Experiment 2 presented claims encountered in the real world, including both true “facts” and common misconceptions, henceforth referred to as “myths”. Claims were followed by explanations that affirmed the facts and corrected the myths. Corrections were either in a non-narrative (NN) or narrative (N) form, and the test was again either immediate or delayed. Thus, Experiment 2 had a 2 × 2 mixed within-between design, with the within-subjects factor of correction type (NN; N) and the between-subjects factor of test delay (immediate; delayed). Fact-affirmation trials acted as fillers outside of this design (although basic affirmation effects are reported).

#### Participants

Experiment 2 used the same recruitment procedures as Experiment 1. Sample size was increased by 10% to allow for the exclusion of participants with more than one initial myth-belief rating of zero (see below).^3^ Participants who participated in Experiment 1 were not allowed to participate in Experiment 2.

A total of 906 participants completed Experiment 2. Retention of participants in the delayed condition was approx. 85%. After applying preregistered exclusion criteria (described in “Results” section), the final sample size for analysis was *N* = 776 (*n* = 385 and *n* = 391 in the immediate and delayed conditions, respectively); the sample comprised 375 men, 393 women, seven nonbinary participants, and one participant of undisclosed gender; mean age was *M* = 33.47 years (SD = 11.44, age range 18–78).

#### Materials

Experiment 2 used eight claims (four myths; four facts). An example myth is “Gastritis and stomach ulcers are caused by excessive stress.” The non-narrative corrections explained the evidence against the claim (e.g., that there is evidence that gastritis and stomach ulcers are primarily caused by the bacterium *Helicobacter pylori* and that this discovery earned the scientists involved a Nobel Prize); the narrative correction detailed the story behind this discovery (e.g., that a scientist drank a broth contaminated with the bacterium to prove his hypothesis, which earned him and his colleague a Nobel Prize). Again, a pilot study confirmed that the narrative corrections were perceived as more story-like and vivid than the non-narrative correction, while being relatively comparable on informativeness and comprehensibility dimensions (see “Appendix” for details). Fact affirmations were of an expository nature similar to the non-narrative corrections. All claims and explanations are provided in “Appendix”.

Each participant received two NN and two N corrections. Assignment of claims (myths M_A-D_) to correction type was counterbalanced, using all six possible combinations (presentation versions V1-6 shown in Table 3); the presentation order of the eight claims (and thus the order of corrections/affirmations as well as narrative conditions) was randomized.

**Table 3:** Presentation versions used in Experiment 2

	M_A_	M_B_	M_C_	M_D_
V1	NN	NN	N	N
V2	NN	N	NN	N
V3	NN	N	N	NN
V4	N	NN	NN	N
V5	N	NN	N	NN
V6	N	N	NN	NN

Versions (V1-6) counterbalanced the assignment of myths (M_A-D_) to conditions (non-narrative correction, NN; narrative correction, N). Assignment of presentation version to participants was randomized, with the constraint that a sixth of participants received each version

Participants rated their belief in each claim on a 0–10 Likert scale immediately after its initial presentation in the study phase (pre-explanation), and again at test (post-explanation). In addition to the second belief rating, the test comprised three inference questions per claim, each requiring a rating of agreement with a statement on a 0–10 Likert scale. The inference questions were designed to measure claim-congruent inferential reasoning (e.g., “Patients with stomach ulcers should avoid any type of stress”). All questions are provided in “Appendix”.

Administration of the survey proceeded as in Experiment 1; the survey file is available at https://osf.io/gtm9z/. The experiment took approximately 10 min. Participants in the immediate condition were reimbursed GBP1.25 (approx. US$1.60) via Prolific; participants in the delayed condition were reimbursed GBP0.60 (US$0.77) for the study phase and GBP0.65 (US$0.83) for the test phase.

#### Procedure

The initial part of the survey was similar to Experiment 1. In the study phase, participants were presented with all eight claims and rated their belief in each. Each rating was followed by an affirmation, or a non-narrative or narrative correction. Materials were again presented for fixed minimum times and the test phase was immediate or delayed (retention interval 1 min vs. 2 days). In the test phase, participants were first presented with the questionnaires of three inference questions per claim. The order of questionnaires was randomized; the order of questions in each questionnaire was fixed (see “Appendix”). Subsequently, participants rated their belief in all claims for a second time. Following the test phase, participants were presented a “data use” question as in Experiment 1.

### Results

Data analysis was preregistered at https://osf.io/akugv; the data are available at https://osf.io/gtm9z/. Analysis adhered to the following procedure: First, exclusion criteria were applied. We excluded data from participants who (a) indicated they do not reside in the USA (*n* = 2); (b) indicated their English proficiency is “fair” or “poor” (*n* = 2); (c) responded to the “data use” question with “No [do not use my data], I really wasn’t paying any attention” (*n* = 1); or (d) responded uniformly (a response SD across all 24 raw rating-scale inference-question responses < 0.5; *n* = 17). To identify inconsistent, erratic responding, we calculated response SD for each set of four test-phase questions and then calculated mean SD across the eight sets. We (e) excluded outliers on this measure, using the interquartile rule with a 2.2 multiplier (i.e., cutoff = Q3 + 2.2 × IQR; *n* = 4). Finally, we excluded participants who (f) had more than one initial myth-belief rating of zero (*n* = 104).

**Fig. 2:**
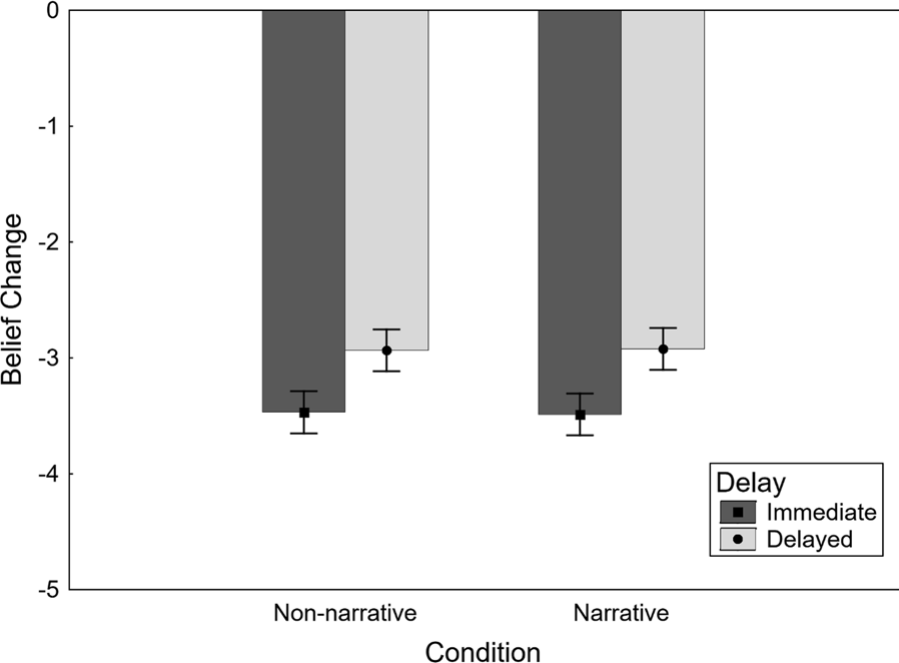
Mean myth-belief-change scores across conditions in Experiment 2; theoretically-possible range was + 10 to − 10. Error bars indicate within-subjects standard error of the mean (Morey 2008)

We calculated four dependent variables relating to myth corrections and fact affirmations, respectively: mean belief-rating change (belief-rating 2−belief-rating 1) for the NN and N conditions, and mean inference scores for the NN and N conditions. We first ran a two-way mixed ANOVA with factors condition (within-subjects) and delay (between-subjects) on myth-belief-change scores (see Fig. 2). This yielded a significant main effect of delay, *F*(1,774) = 10.78, MSE = 10.90, *η*_p_^2^ = .014, *p* = .001, indicating greater belief change in the immediate test. Both the main effect of condition and the interaction were nonsignificant, *F* < 1. The planned contrasts of NN versus N conditions at either delay were also nonsignificant, *F* < 1. Mean belief change for facts was *M* = 3.66 (SD = 2.39) in the immediate test and *M* = 3.87 (SD = 2.35) in the delayed test. Both values differed significantly from zero, *t*(384/390) > 30.05, *p* < .001, but not from each other, *F*(1,774) = 1.47, MSE = 5.62, *η*_p_^2^ = .002, *p* = .225.

**Fig. 3:**
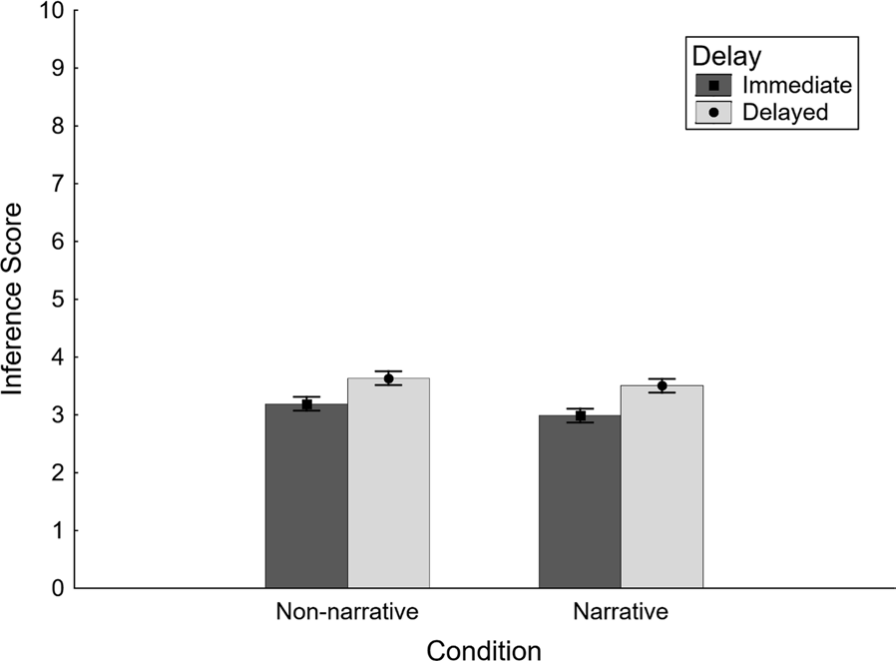
Mean myth inference scores across conditions in Experiment 2. Greater values indicate greater misinformation reliance. Error bars indicate within-subjects standard error of the mean (Morey 2008)

We then ran the same two-way mixed ANOVA on inference scores (see Fig. 3). This yielded a significant main effect of delay, *F*(1,774) = 8.52, MSE = 10.44, *η*_p_^2^ = .011, *p* = .004, indicating lower scores in the immediate test. There was also a marginal main effect of condition, *F*(1,774) = 3.98, MSE = 2.65, *η*_p_^2^ = .005, *p* = .046, suggesting lower scores in the narrative condition (*F* < 1 for the interaction). However, the core planned NN versus N contrast was nonsignificant in both the immediate test, *F*(1,774) = 2.90, *η*_p_^2^ = .004, *p* = .089, and the delayed test, *F*(1,774) = 1.25, *η*_p_^2^ = .002, *p* = .264. Mean inference scores for facts were *M* = 7.77 (SD = 1.18) in the immediate test and *M* = 7.65 (SD = 1.26) in the delayed test; this was not a significant difference, *F*(1,774) = 1.95, MSE = 1.49, *η*_p_^2^ = .003, *p* = .163.

To complement this frequentist analysis (and to quantify evidence in favor of the null), we ran Bayesian *t* tests comparing NN and N in both delay conditions. We first did this with belief-change scores: In the immediate condition, this returned a Bayes Factor of BF_01_ = 17.37; in the delayed condition, we found BF_01_ = 17.55. This means that the data are approx. 17 times more likely under the null hypothesis of no difference between narrative conditions, which is strong evidence in favor of the null (Wagenmakers et al. 2018). We then tested inference scores: In the immediate condition, this returned BF_01_ = 3.70; in the delayed condition, we found BF_01_ = 9.92. This means that the data are approx. 4–10 times more likely under the null hypothesis of no difference between narrative conditions; this constitutes moderate evidence in favor of the null (Wagenmakers et al. 2018).

Furthermore, to take initial belief levels into account more generally, we ran linear mixed-effects models. Presentation version and participant ID (nested in presentation version) were included as random effects, and experimental condition, delay, their interaction, and initial belief were fixed effects, predicting test-phase myth-belief ratings and inference scores. As with the ANOVAs, we did this for the full 2 × 2 design, but also separately for each delay condition, thus with only condition and initial belief as fixed effects. Results are provided in Table 4. In the full design, myth belief at test (belief rating 2) was predicted significantly by delay and the initial belief rating 1. Inference scores were likewise predicted significantly by delay and belief rating 1. In both cases, experimental condition was not a significant predictor. When analyses were restricted to the immediate and delayed conditions, respectively, the results were comparable: only initial belief was a significant predictor of test-phase belief, and experimental condition was not a significant predictor.

**Table 4:** Linear mixed-effects modeling results in Experiment 2

Predictor	Full design					Immediate					Delayed				
Belief Rating 2	|β|	SE	df	|t|	p	|β|	SE	df	|t|	p	|β|	SE	df	|t|	p
Condition	0.05	0.13	2315	0.36	.718	0.05	0.12	1147	0.40	.693	0.05	0.20	1167	0.35	.725
Delay	0.54	0.19	1276	2.82	.005	–	–	–	–	–	–	–	–	–	–
Condition × Delay	< 0.01	0.19	2315	0.01	.990	–	–	–	–	–	–	–	–	–	–
Belief Rating 1	0.24	0.02	2779	14.40	< .001	0.23	0.02	1356	10.19	< .001	0.26	0.03	1419	10.12	< .001
**Inference Scores**															
Condition	0.19	0.12	2318	1.64	.102	0.19	0.11	1149	1.72	.085	0.11	0.12	1168	0.90	.371
Delay	0.44	0.18	1222	2.51	.012	–	–	–	–	–	–	–	–	–	–
Condition × Delay	0.08	0.16	2318	0.50	.616	–	–	–	–	–	–	–	–	–	–
Belief Rating 1	0.25	0.01	2739	16.76	< .001	0.25	0.02	1340	12.12	< .001	0.25	0.02	1398	11.60	< .001

### Discussion

Experiment 2 tested whether corrections targeting real-world misconceptions are more effective if they are provided in a narrative versus non-narrative format. The results were clearcut: While corrections effected substantial belief change, which was only moderately reduced by a 2-day delay, there was no difference between narrative and non-narrative conditions. When assessing myth beliefs through more indirect post-correction inference questions, there was likewise little evidence of a narrative benefit: While the main effect of condition was marginally significant in the omnibus analysis, the core contrasts of narrative and non-narrative conditions at each delay were nonsignificant. Moreover, the Bayesian analyses consistently provided support in favor of the null hypothesis of no difference between narrative and non-narrative conditions.

Experiments 1 and 2 therefore provide evidence that narrative corrections do not promote more event-memory updating or knowledge revision than non-narrative corrections. These results suggest that the narrative format does not facilitate comprehension, integration, or retrieval of the correction. However, it is possible that the narrative format produces corrective benefit in situations where there might be some opposition to the content of the correction, given past work showing that narratives reduce resistance persuasive messages relative to non-narrative counterparts (see Green and Brock 2000; Krakow et al. 2018; Slater and Rouner 1996). Experiment 3 tested this possibility.

## Experiment 3

Narratives reduce counterarguing relative to non-narrative messages (Green and Brock 2000; Slater and Rouner 1996). One might, therefore, suggest that narrative-format corrections should be particularly effective (relative to non-narrative corrections) when the content of a message challenges a person’s worldview. Experiment 3 examined the effect of messages addressing more controversial, real-world claims, where a correction can be expected to be worldview-inconsistent for the majority of participants. It therefore enabled a more focused test of underlying process, as well as an examination of the effect of corrective message format in a context of practical significance. Specifically, two myths expected to resonate with more conservative participants were used, and only people who identified as conservative were recruited as participants.

### Method

Experiment 3 presented claims encountered in the real world, including both facts and myths, which were followed by affirmations and corrections. Corrections were again either non-narrative (NN) or narrative (N), and the test was immediate or delayed. Thus, Experiment 3 had a 2 × 2 mixed within-between design, with the within-subjects factor of correction type (NN; N) and the between-subjects factor of test delay (immediate; delayed). Fact-affirmation trials acted as fillers outside of this design (although basic affirmation effects will be reported).

#### Participants

Target sample size was the same as in Experiment 2, but we used a sample of adult US residents who indicated that they identify as politically conservative, recruited via Prolific.^4^ Participants who participated in Experiment 1 or 2 were not allowed to participate in Experiment 3. Similar to Experiment 2, oversampling (again, by 10%) was applied to account for exclusions of participants with low initial myth-belief ratings. Due to a large number of exclusions based on preregistered criteria, minor resampling was used to achieve the required sample size, as per the preregistered plan.

Initially, a total of 953 participants completed Experiment 2. Retention of participants in the delayed condition was greater than expected (approx. 93%). After applying preregistered exclusion criteria (described in “Results” section), 725 participants remained, with *n* = 345 in the immediate condition and *n* = 380 in the delayed condition. As the number of participants in the immediate condition dropped below the minimum prespecified cell size of *n* = 352, we resampled, following the preregistered plan, obtaining an additional eight participants in the immediate condition. The final sample size for analysis was *N* = 733 (*n* = 353 and *n* = 380 in the immediate and delayed conditions, respectively); the sample comprised 435 men, 297 women, and one participant of undisclosed gender; mean age was *M* = 38.47 years (SD = 14.22, age range 18–84).

#### Materials

Experiment 3 used four claims (two myths; two facts). One myth was “Humans are made to eat red meat; it should be part of every person’s diet.” The other was “Children of homosexual parents have more mental health issues.”^5^ The non-narrative corrections explained the evidence suggesting that the claim is false (e.g., evidence that eating red meat on a regular basis will shorten people’s lifespans and that replacing it with other foods could lower mortality risk by 7 to 19%); the narrative corrections contained the same facts but were presented as a quote from someone to whom the claim is directly relevant (e.g., a meat-lover explaining how their daughter pleaded with them to eat less red meat and rotate in other foods). Again, a pilot study confirmed that the narrative corrections were perceived as more story-like and vivid than the non-narrative correction, while being relatively comparable on informativeness and comprehensibility dimensions (see “Appendix” for details).^6^ Fact affirmations were expository in nature, similar to the non-narrative corrections. All claims and explanations are provided in “Appendix”. Each participant received one NN and one N correction. The correction type applied to each myth was counterbalanced, and presentation order of the claims was randomized. Measures were implemented as in Experiment 2 (an example inference question is “To maintain a healthy diet, people should regularly consume red meat”). All questions are provided in “Appendix”.

Administration of the survey proceeded as in Experiment 2; the survey file is available at https://osf.io/gtm9z/. The experiment took approximately 8 min. Participants in the immediate condition were reimbursed GBP1 (approx. US$1.30) via Prolific; participants in the delayed condition were reimbursed GBP0.45 (US$0.60) for the study phase and GBP0.55 (US$0.70) for the test phase.

#### Procedure

The procedure was identical to Experiment 2 (with the exception that participants viewed only four claims).

### Results

Data analysis was preregistered at https://osf.io/5yxse. Analysis adhered to the same procedure as Experiment 2: First, exclusion criteria were applied. We excluded data from participants who (a) indicated they do not reside in the USA (*n* = 2); (b) indicated their English proficiency is “fair” or “poor” (*n* = 0); (c) responded to the “data use” question with “No [do not use my data], I really wasn’t paying any attention” (*n* = 1); or (d) responded uniformly (a response SD across all 12 raw rating-scale inference-question responses < 0.5; *n* = 24). To identify inconsistent, erratic responding, we calculated response SD for each set of four test-phase questions, then calculated mean SD across the four sets. We (e) excluded outliers on this measure, using the interquartile rule (i.e., cutoff = Q3 + 2.2 × IQR; *n* = 6). Finally, we excluded participants with any initial myth-belief rating < 1, or both initial myth-belief ratings < 2 (*n* = 195).^7^

**Fig. 4:**
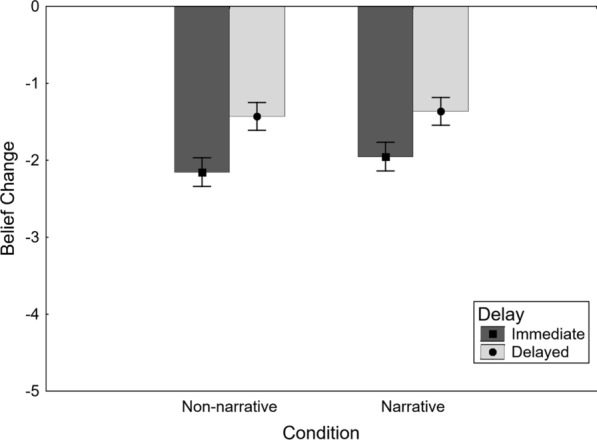
Mean myth-belief-change scores across conditions in Experiment 3; theoretically-possible range was + 10 to − 10. Error bars indicate within-subjects standard error of the mean (Morey 2008)

We calculated mean belief-rating change (belief-rating 2−belief-rating 1) for the NN and N conditions, and mean inference scores for the NN and N conditions. We first ran a two-way mixed ANOVA with factors condition (within-subjects) and delay (between-subjects) on myth-belief-change scores (see Fig. 4). This yielded a significant main effect of delay, *F*(1,731) = 16.23, MSE = 9.71, *η*_p_^2^ = .022, *p* < .001, indicating greater belief change in the immediate test. Both the main effect of condition and the interaction were nonsignificant, *F* ≤ 1.06. The planned contrasts of NN versus N conditions at either delay were also nonsignificant, *F* ≤ 1.16. Mean belief change for facts was *M* = 1.80 (SD = 1.86) in the immediate test and *M* = 1.46 (SD = 1.93) in the delayed test. Both values differed significantly from zero, *t*(352/379) > 14.71, *p* < .001, and also from each other, *F*(1,731) = 5.90, MSE = 3.61, η_p_^2^ = .008, p = .015.

**Fig. 5:**
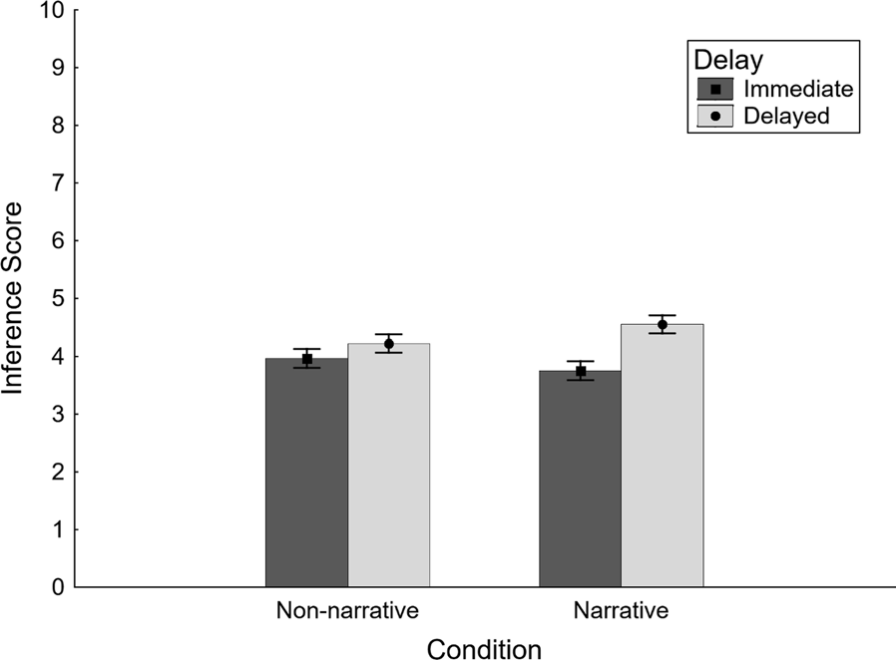
Mean myth inference scores across conditions in Experiment 3. Greater values indicate greater misinformation reliance. Error bars indicate within-subjects standard error of the mean (Morey 2008)

We then ran the same two-way mixed ANOVA on inference scores (see Fig. 5). This yielded a significant main effect of delay, F(1,731) = 9.49, MSE = 10.62, η_p_^2^ = .013, p = .002, indicating lower scores in the immediate test. There was no main effect of condition, F < 1, but a significant delay × condition interaction, F(1,731) = 5.78, MSE = 4.68, η_p_^2^ = .008, p = .016. The core planned NN versus N contrast was nonsignificant in the immediate test, F(1,731) = 1.73, η_p_^2^ = .002, p = .188. The contrast was significant in the delayed test, F(1,731) = 4.40, η_p_^2^ = .006, p = .036; however, this effect was in the opposite direction than predicted, with lower inference scores in the non-narrative condition. Mean inference score for facts were M = 7.87 (SD = 1.53) in the immediate test and M = 7.92 (SD = 1.46) in the delayed test; this difference was not significant, F < 1.

As in Experiment 2, we ran complementary Bayesian t tests comparing the effect of correction format in both delay conditions, separately. We first examined the effect on belief-change scores: In the immediate condition, this returned a Bayes Factor of BF_01_ = 9.39; in the delayed condition, we found BF_01_ = 16.25. These results provide moderate to strong evidence in favor of the null. We then tested the effect on inference scores: In the immediate condition, this returned BF_01_ = 7.03, providing moderate evidence in favor of the null; in the delayed condition, we found BF_01_ = 2.03, which provides only anecdotal evidence, but also in favor of the null (Wagenmakers et al. 2018).^8^

As in Experiment 2, we ran linear mixed-effects models to take initial myth belief into account. Results are provided in Table 5. In the full design, delay and the initial belief rating 1 predicted test-phase myth belief (belief rating 2). Inference scores were predicted only by belief rating 1. In both cases, experimental condition was not a significant predictor. Analyses restricted to the immediate and delayed conditions, respectively, yielded comparable results: Initial myth belief was a significant predictor of test-phase belief and experimental condition was not.

**Table 5:** Linear mixed-effects modeling results in Experiment 3

Predictor	Full design					Immediate					Delayed				
Belief Rating 2	|β|	SE	df	|t|	p	|β|	SE	df	|t|	p	|β|	SE	df	|t|	p
Condition	0.07	0.16	717	0.45	.651	0.07	0.16	337	0.47	.639	0.15	0.16	377	0.91	.365
Delay	0.64	0.20	1308	3.29	.001	–	–	–	–	–	–	–	–	–	–
Condition × Delay	0.07	0.23	718	0.32	.752	–	–	–	–	–	–	–	–	–	–
Belief Rating 1	0.57	0.03	1446	21.55	< .001	0.57	0.04	686	15.18	< .001	0.57	0.04	754	15.28	< .001
Inference Scores															
Condition	0.08	0.15	720	0.51	.607	0.06	0.15	339	0.38	.707	0.26	0.15	377	1.72	.087
Delay	0.34	0.18	1328	1.89	.059	–	–	–	–	–	–	–	–	–	–
Condition × Delay	0.32	0.21	720	1.52	.130	–	–	–	–	–	–	–	–	–	–
Belief Rating 1	0.46	0.02	1453	18.47	< .001	0.53	0.04	702	15.06	< .001	0.40	0.03	752	11.54	< .001

### Discussion

Experiment 3 tested whether narrative corrections would be more effective than non-narrative corrections when debunking worldview-consistent misconceptions. It has been argued that efforts to correct such worldview-supported beliefs are potentially less effective (Lewandowsky et al. 2012; Nyhan and Reifler 2010; but see Ecker et al. 2020; Swire-Thompson et al. 2020; Wood and Porter 2019). Therefore, identifying ways to successfully reduce belief in worldview-consistent misinformation may be particularly valuable. The corrections applied in this study did not change beliefs as much as in Experiment 2, presumably due to the effect of worldview. More importantly, narrative corrections were not more effective in reducing beliefs than non-narrative corrections. While there was a small effect of correction format on inference scores in the delayed condition, this effect indicated more misinformation reliance in the narrative condition compared to the non-narrative condition. However, we do not interpret this finding as suggesting that narrative corrections are inferior, given that in the pilot study the non-narrative corrections in Experiment 3 were rated as slightly more informative than the narrative corrections.

## General discussion

In three experiments, we tested the hypothesis that narrative corrections are more effective than non-narrative corrections at reducing misinformation belief and reliance. We observed a range of findings that conform to previous research: We found a small continued influence effect in Experiment 1; correction effects were generally larger in the immediate versus delayed tests; and post-correction belief ratings and inference scores were predicted by test-phase delay and initial belief ratings in the mixed-effects modeling. However, with regard to the core hypothesis of a narrative benefit, results were clearcut: The narrative versus non-narrative format of the correction had no impact on the correction’s effectiveness, in terms of either misinformation belief change or inferential reasoning scores.

Theoretically, we proposed that narrative corrections might be more effective due to (1) enhanced processing of the correction, as stories tend to result in stronger emotional involvement and transportation (e.g., Green and Brock 2000; Hamby et al. 2018); (2) suppression of counterargument generation, caused by immersion in the narrative (e.g., Green and Brock 2000; Slater and Rouner 1996); or (3) enhanced retrieval, resulting either from a more vivid memory representation or the availability of potent retrieval cues relating to the narrative structure (e.g., Bruner 1986; Graesser and McNamara 2011). Our results provided no support for these proposals. Instead, results suggest that the narrative versus non-narrative format does not matter for misinformation debunking, as long as corrections are easy to comprehend and contain useful, relevant, and credible information (see Lewandowsky et al. 2020; Paynter et al. 2019). An alternative interpretation is that a narrative format potentially does have benefits, but that these were offset in our study by the narrative elements distracting from the correction’s core message. However, given that the null effect of correction format was replicated across three experiments with substantial differences in materials, we prefer the simpler interpretation that the format of a correction (narrative or non-narrative) has little effect on a corrective message’s efficacy.

This, in turn, suggests that anecdotal evidence for the superiority of narrative corrections may have arisen from confounds between the narrative versus non-narrative correction format and other elements such as the amount, quality (i.e., persuasiveness), or novelty of information provided. For example, past work shows that effective corrections contain greater detail (e.g., Chan et al. 2017; Swire et al. 2017) or feature a causal alternative explanation (e.g., Ecker et al. 2010; Johnson and Seifert 1994). In the current work, we held constant not only the amount but also the type of corrective details (i.e., causal explanations) included in each correction.

The present study contributes broadly to the substantial body of research comparing the persuasive efficacy of different message formats, which has yielded conflicting results: While some work shows that narratives and non-narratives are equally persuasive (Dunlop et al. 2010), other findings suggest that one format is superior to the other (Greene and Brinn 2003; Ratcliff and Sun 2020; Zebregs et al. 2015a). These diverging results suggest that a line of inquiry directed toward identifying when message format makes a difference in both initial and corrective persuasion may be fruitful. For instance, the claim and corrective contexts examined in the current work generally mirrored those that are encountered in news media. A recent meta-analysis (Freling et al. 2020) identified message content as a determinant of the persuasive efficacy of message format, such that narrative-based messages are more persuasive when emotional engagement is high (as when focal content involves a severe threat to health or oneself). It is similarly possible that the format of a corrective message may matter when the topic is emotionally engaging, but not in more generally informative scenarios such as those examined in the present work. In support of this position, it has been suggested that personal experiences of people affected by COVID-19 can serve to reduce misconceptions about the pandemic (Mheidly and Fares 2020).

A challenge in comparing the persuasive (or corrective) efficacy of narrative versus non-narrative messages lies in operationalizing message format in a way that is true to their conceptual definition but that does not also introduce confounds (van Krieken and Sanders 2019). While we carefully attempted to minimize confounds in the present work, there are several limitations. In fact, our efforts to make narrative and non-narrative messages as equivalent as possible on the dimensions of length and featured content may obscure differences on these dimensions that occur naturally. Further, while steps were taken to enhance external validity in the current work, participants in online experiments are not representative of the public at large, and engagement with the materials in such experiments is always somewhat contrived. Specifically, experimental procedures involving corrections are subject to demand characteristics, and participants are incentivized to pay attention to all presented information. Part of stories’ persuasive potential lies in their ability to attract and retain attention, which is particularly important in the modern media environment. Thus, future work examining the effect of message format on debunking efforts in a field context is warranted. Stories that are cocreated with the audience may be useful in addressing misinformation, particularly in contexts characterized by limited access to or engagement with high-quality, fact-oriented information sources. Moreover, approaches that jointly present evidence and narrative elements, such as narrative data visualization (e.g., Dove and Jones 2012), might provide a particularly promising approach for future interventions. What we can conclude from the present study, however, is that the narrative format, in itself, does not generally (i.e., under all conditions) produce an advantage when it comes to misinformation debunking.

## Figures and Tables

**Fig. 6:**
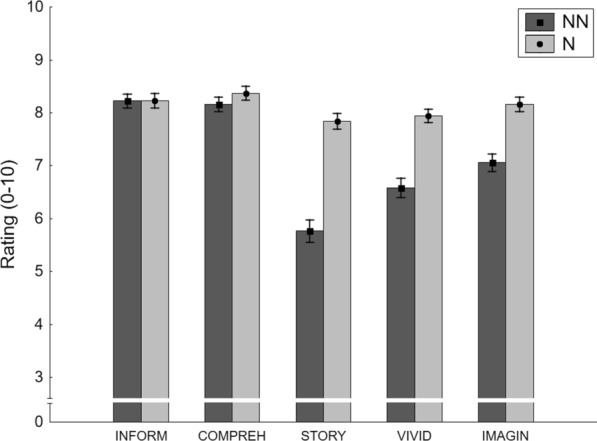
Ratings of non-narrative (NN) and narrative (N) event-report corrections on informativeness (INFORM), comprehensibility (COMPREH), story-ness (STORY), vividness (VIVID), and imaginability (IMAGIN) in the Experiment 1 Pilot. Error bars indicate within-subjects standard error of the mean

**Fig. 7:**
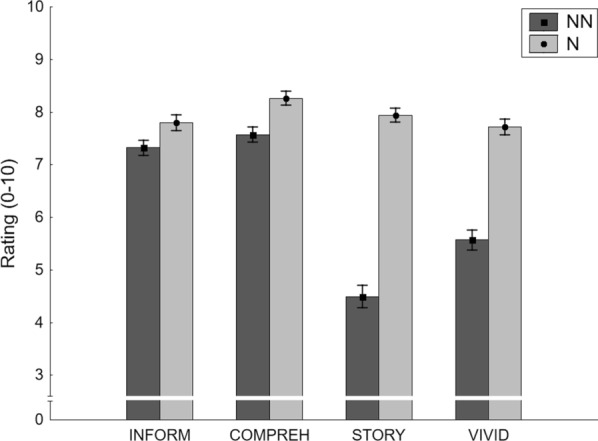
Ratings of non-narrative (NN) and narrative (N) myth corrections on informativeness (INFORM), comprehensibility (COMPREH), story-ness (STORY), and vividness (VIVID) in the Experiment 2 Pilot. Error bars indicate within-subjects standard error of the mean

**Fig. 8:**
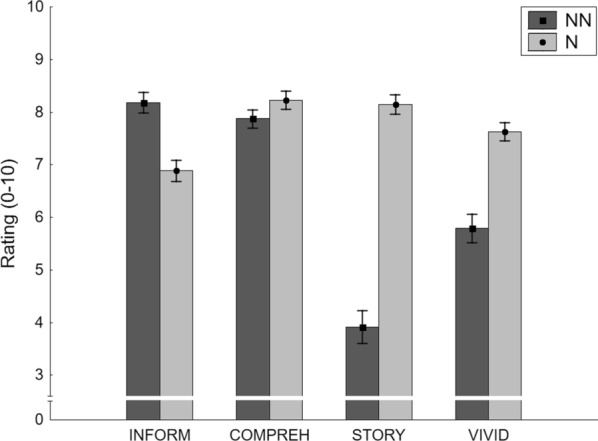
Ratings of non-narrative (NN) and narrative (N) myth corrections on informativeness (INFORM), comprehensibility (COMPREH), story-ness (STORY), and vividness (VIVID) in the Experiment 3 Pilot. Error bars indicate within-subjects standard error of the mean

## Data Availability

All data and survey files (which include the materials) are available on the Open Science Framework Web site. For convenience, all materials are additionally provided in “Appendix”.

## Appendix

### Experiment 1

**Event reports.** On average, the non-narrative corrections contained in the event reports had 111 words, with a Flesch reading ease (FRE) score of 49.23 and a Flesch–Kincaid grade level (FKGL) of 11.6. Narrative corrections had 111.25 words, with a reading ease score of 43.05 and a grade level of 11.73.

***Report A: Wildfire***. (356–359 words)

Article 1.

VANCOUVER—Firefighters in British Columbia have been battling a wildfire that raged out of control in the state’s^9^ South-East overnight. The fire came dangerously close to homes in the town of Cranbrook, but it is believed that no damage was caused to property. [David Karle of the BC Wildfire Service indicated that authorities were looking into the cause of the fire, with early evidence suggesting that the fire had been deliberately lit. Despite extensive campaigns, arson remains a significant problem in the region, and a leading cause of wildfires globally.]^10^ Emergency services were still working tirelessly this morning to extinguish the flames, but were confident that the location of the remaining fire was unlikely to pose any further threat to local communities. (Word Count [WC] = 121; Flesch Reading Ease [FRE] = 40.3; Flesch–Kincaid Grade Level [FKGL] = 13.6)

Article 2.

VANCOUVER—After working throughout the day, firefighters have managed to bring a wildfire in the South-East of British Columbia under control. There have been no reported casualties or damage to property, with most land damage occurring in rural fringe areas and nearby forest reserves. The suspected burn area is estimated to be roughly 10,000 hectares. (WC = 54; FRE = 36.5; FKGL = 12.6)

Non-narrative correction: It is now clear that the fire was caused by a power line from a fallen power pole. The power pole was in a condition that was substantially weakened due to general rot and severe damage caused by the growth of a colony of termites. The cause of the fire was announced earlier today by Cranbrook Fire and Emergency Services based on new evidence that emerged from a detailed additional investigation of the ignition zone (the area where the fire had started). This investigation took place shortly after the fire in that area had been extinguished. A power line from the broken pole had made contact with the ground and started the fire, after the power pole had fallen. (WC = 119; FRE = 58.2; FKGL = 11)

Narrative correction: An additional investigation by Fire Chief Warren Linnell uncovered the true fire cause: a power line from a fallen power pole. Linnell, a 20-year veteran of the Cranbrook Fire and Emergency Services, was skeptical of initial claims about the fire’s cause: “I’ve seen a lot of fires, and determining the cause of any fire always requires thorough investigation.” Deciding to explore further, Linnell waded through the ignition zone and discovered a power pole that had snapped. Peering closely, he noticed rot and severe termite damage throughout the pole. Then, he noticed the broken power line. When he saw that it had melted on the ground, he concluded that the broken power line ignited leaf litter around the broken pole, starting the fire. (WC = 122, 1.03 ratio; FRE = 51.9; FKGL = 11.1)

Casey Haas, a resident of Cranbrook, expressed her relief that no one had been injured by the fire, saying she felt lucky that they had avoided disaster, and that her beloved ponies Tom and Jerry had survived unharmed. Even so, she felt it was important for residents of the community to work together to ensure they are prepared for potential future disasters. (WC = 62; FRE = 43; FKGL = 14.9)

***Report B: Spike in seizures.*** (347–348 words)

Article 1.

BRISBANE—An unprecedented spike in seizures leading to hospital admissions has been reported in North Queensland (Australia). Over the past month, 17 children were assessed at Townsville Hospital, with roughly half being admitted for observation and in-patient treatment. According to the hospital, these are unusual numbers for the regional town, which has a population of 180,000. [The spike in seizures has been linked to the introduction of a new compound vaccine, offered to children in the region, which combines the polio and chicken pox (varicella) vaccines. It was hoped the new vaccine would increase the immunization rate against chicken pox, as part of an active push to completely eradicate the disease in Australia. However, seizures can be a side effect of vaccination, and administration of the new vaccine has been suspended.] At this stage, none of the seizures have been life-threatening, although three children remain in hospital under close surveillance. (WC = 149; FRE = 36.4; FKGL = 13.4)

Article 2.

BRISBANE—All children affected by a recent spike in seizures in North Queensland have now returned home to their families. While several new cases have been reported, none has required hospitalization. (WC = 30; FRE = 50.6; FKGL = 9.9)

Non-narrative correction: The spike in seizures recently seen at a North-East Australian hospital has now been linked to the Kuta virus, a virus most commonly seen in rural parts of South East Asia. The increase in seizures occurred at the same time as an increase in the level of mosquito activity in the region. Evidence of the Kuta virus was present in all examined blood samples tested. The virus is known to cause seizures in children, although it is not usually present in Australia. According to experts, the unusually high temperatures seen in the region over the past months could have contributed to the spread of the virus. (WC = 106; FRE = 52; FKGL = 11.2)

Narrative correction: Health authorities have now linked the spike in seizures to the Kuta virus. Dr. Katherine Hopkins from Townsville Hospital noticed a report about high mosquito activity in the region. She became curious whether there was any connection to the seizures. Running additional tests on patients’ blood, she found evidence of the Kuta virus, which is known to cause seizures, in all samples. “I was surprised at first, because the virus is usually not present in Australia” Dr. Hopkins said, “so I called my colleague, who is an epidemiologist.” The epidemiologist, Dr. David Chang, confirmed that the unusually high temperatures likely allowed the virus to spread. (WC = 105, .99 ratio; FRE = 44.8; FKGL = 11.3)

Locals Daniel and Tiarne Corner explained that their 5-year-old son Toby had just been released from hospital and expressed their gratitude to the hospital’s staff: “It was so scary when the seizures started, out of the blue. The nurses and doctors took such good care of us; they are amazing. We are so glad it’s over, and can’t wait to go home.” (WC = 64; FRE = 71.5; FKGL = 8.5)

***Report C: Plane crash.*** (362 words)

Article 1.

MANCHESTER—A small business jet en route to the German town of Rostock crashed on Monday morning, minutes after takeoff from Manchester Airport. The two-engine Zephyr ZX crashed in a field near the town of Failsworth, killing all eleven people—eight passengers and three crew—on board. The passengers are believed to be the executives of Manchester-based technology start-up 3RTec. [Based on initial evidence and witness reports, the plane stalled after hitting a drone that was flying in the area. Despite regulations, drones flying near airports have been identified as a significant but difficult-to-eliminate threat to air travel safety.] Witnesses described that they heard a loud explosion and saw a plume of black smoke when the aircraft hit the ground. “A few hundred yards further down, and it would have struck my house,” local resident Liesel Mason noted. “It was frightening. I really feel for the victims, it must have been terrifying.” (WC = 151; FRE = 56.4, FGKL = 9.5)

Article 2.

MANCHESTER—The Manchester business community is still in shock after Monday’s plane crash, which killed eleven people, including the entire executive team of local tech company 3RTec. Alice Crane, the company’s HR manager, explained that staff are absolutely devastated. “There are no words,” Ms. Crane stated. “We just don’t feel like this is real.” (WC = 54; FRE = 54.5; FKGL = 8.9)

Non-narrative correction: The plane crash near Manchester has now been ruled the result of a technical failure of the machinery inside the plane. In a statement put out by the UK’s Civil Aviation Authority, it was revealed that the plane contained a manufacturing flaw specific to Zephyr ZX aircraft manufactured recently in the company’s Aberdeen plant. One of the engines’ thrust reversers accidentally deployed shortly after takeoff at an altitude of 3000 ft. A thrust reverser is part of an engine; it changes the direction of airflow and is used by pilots to slow a plane down during or after landing. Deployment of the thrust reverser caused the plane to bank to the right and enter a high-speed dive. (WC = 118; FRE = 49.9; FKGL = 11.1)

Narrative correction: An additional investigation has revealed that the devastating plane crash near Manchester was caused by a technical failure. Investigator Sharon Williams from the UK’s Civil Aviation Authority said: “I became suspicious after learning that the aircraft had been manufactured in Zephyr’s Aberdeen plant. A concerned Zephyr employee previously confided in me that a manufacturing flaw had been detected in this plant. The company was trying to downplay it.” Williams’ team investigated and found evidence that one of the engines’ thrust reverser had malfunctioned. Williams explained: “A thrust reverser acts like a brake. This one deployed shortly after take-off at an altitude of 3000 ft. This caused the plane to bank to the right and enter a high-speed dive.” (WC = 118, 1.00 ratio; FRE = 41.3; FKGL = 11.1)

While this was the third fatal aviation accident in the UK in the past month, flying continues to be a very safe mode of transportation. The overwhelming majority of aviation fatalities involve small, private airplanes, and not large commercial airliners. (WC = 40; FRE = 36.3; FKGL = 13.1)

***Report D: Salmonella outbreak.*** (318–320 words)

Article 1.

ALBUQUERQUE—More than a hundred people have fallen ill—and a dozen have been hospitalized—after a salmonella outbreak in New Mexico. Victims had dined at several restaurants in the greater Albuquerque area. [The outbreak has been traced back to a local food factory, where it is believed the failure of sterilization equipment is to blame for the food poisoning. The factory, which produces mayonnaise and other condiments for local restaurants, has stopped production and recalled products.] An estimated 1.2 million salmonella cases occur in the USA annually. [While many cases are related to food hygiene in the home, larger outbreaks are often linked to technical issues during food production.] While the current outbreak in New Mexico is significant, the largest outbreak in US history in 2008 saw more than 1000 people fall ill in Texas and several other states. (WC = 139; FRE = 39.3; FKGL = 12.6)

Article 2.

ALBUQUERQUE—The total number of victims who have fallen ill in the New Mexico salmonella outbreak has risen to 137. While most victims are recovering well, a 79-year-old North Valley man had to be admitted into intensive care and is in a critical condition. (WC = 43; FRE = 42.2; FKGL = 12.8)

Non-narrative correction: The outbreak in the Albuquerque processing plant has now been linked to intentional food contamination. This means that food had become corrupted with another substance during processing. The sterilization equipment at the factory was found to work adequately and reliably heat all foods to 170 degrees Fahrenheit, which is a high enough temperature to destroy any biological contaminants. However, a review of the CCTV footage from the factory showed a male employee in the packaging department of the factory tampering with a product as it was bottled. It appears the employee’s motive to do so was revenge for poor treatment of staff. (WC = 102; FRE = 36.8; FKGL = 13.1)

Narrative correction: An additional investigation by inspector Stephanie Hill from the Food Safety Authority has uncovered that the outbreak was the result of intentional food contamination. During her inspection of the Albuquerque factory, Hill found that the sterilization equipment worked adequately, heating foods to the required 170 degrees Fahrenheit. “This seemed suspicious, so I decided to review the CCTV footage,” Hill described. What she found shocked her: the tapes showed an employee contaminating a product as it was bottled. When confronted, the employee exploded with rage, describing his desire to ruin the company as revenge for his boss’ cruel treatment of staff. (WC = 100, .98 ratio; FRE = 34.2; FKGL = 13.4)

All restaurants remain open for business and are preparing for the upcoming Albuquerque Restaurant Week, an annual event that celebrates the local food scene. Curious patrons can expect fiery and creative meals, with many special offers. (WC = 36; FRE = 38.1; FKGL = 12.4)

**Test questionnaires.**

***Report A.***

1. The fire came close to the town of Cranbrook/Kimberley/Lumberton/Bull River
2. “Devastating wildfire intentionally lit” would be an appropriate headline for the report. 0 (Strongly Disagree)–10 (Strongly Agree)
3. Malicious intent contributed to the fire. 0 (Strongly Disagree)–10 (Strongly Agree)
4. The person responsible for the wildfire should be identified and charged. 0 (Strongly Disagree)–10 (Strongly Agree)
5. The local government should invest in measures to prevent arson. 0 (Strongly Disagree)–10 (Strongly Agree)
6. Local residents should be particularly vigilant against potential arsonists. 0 (Strongly Disagree)–10 (Strongly Agree)
7. What do you think caused the wildfire? Arson/Lightning/Power line/None of the above

***Report B.***

1. Which Australian state was affected by the seizures? Queensland/New South Wales/Victoria/Tasmania
2. “New vaccine leads to seizures, hospitalizations” would be an appropriate headline for this report. 0 (Strongly Disagree)–10 (Strongly Agree)
3. Insufficient safety tests by pharma companies contributed to the spike in seizures. 0 (Strongly Disagree)–10 (Strongly Agree)
4. There should be repercussions for the person who approved the vaccine trial. 0 (Strongly Disagree)–10 (Strongly Agree)
5. The government should implement more stringent safety tests of vaccines to prevent such incidents in the future. 0 (Strongly Disagree)–10 (Strongly Agree)
6. Based on what happened, parents should be particularly skeptical of newly introduced compound vaccines. 0 (Strongly Disagree)–10 (Strongly Agree)
7. What do you think caused the spike in seizures? Vaccine/Lead poisoning/Virus/None of the above

***Report C.***

1. How many people were killed in the crash? 11/16/20/25
2. “Drone downs plane, killing all aboard” would have been an appropriate headline for the report. 0 (Strongly Disagree)–10 (Strongly Agree)
3. A drone collision contributed to the plane crash. 0 (Strongly Disagree)–10 (Strongly Agree)
4. The person flying the drone should be identified and charged with manslaughter. 0 (Strongly Disagree)–10 (Strongly Agree)
5. Following the incident, policies regarding drone usage around airports should be reviewed. 0 (Strongly Disagree)–10 (Strongly Agree)
6. Based on this event, drone-detection hardware should be made mandatory on all aircraft. 0 (Strongly Disagree)–10 (Strongly Agree)
7. What do you think caused the plane crash? Drone strike/Bad weather/Technical fault/None of the above

***Report D.***

1. How many people fell ill during the New Mexico salmonella outbreak? About 50/More than 100/More than 250/More than 500
2. “Equipment failure causes salmonella outbreak” would be an appropriate headline for this report. 0 (Strongly Disagree)–10 (Strongly Agree)
3. A technical issue contributed to the outbreak. 0 (Strongly Disagree)–10 (Strongly Agree)
4. There should be repercussions for the factory staff responsible for equipment maintenance and testing. 0 (Strongly Disagree)–10 (Strongly Agree)
5. Based on this incident, food factories should implement more stringent safety tests of sterilization equipment to prevent such incidents in the future. 0 (Strongly Disagree)–10 (Strongly Agree)
6. The affected company should consider investing in more reliable sterilization equipment. 0 (Strongly Disagree)–10 (Strongly Agree)
7. What do you think caused the outbreak? Equipment failure/Restaurant hygiene/Intentional tampering/None of the above

**Pilot Study.** One hundred US-based MTurk workers (min. 5000 so-called Human Intelligence Tasks [HITs] completed with 98% + approval rate) were recruited to rate the non-narrative and narrative corrections of all event reports. One participant was excluded due to uniform responding (SD = 0), leaving N = 99 participants (M_age_ = 40.44 years; age range 20–79; 51 males, 46 females, 2 of unspecified gender).

All reports were presented in randomized order. For each report, participants read both corrections, also in randomized order. They were asked to rate each correction on informativeness (“How informative is the correction?”), comprehensibility (“How easy to understand is the correction?”), story-ness (“How story-like is the correction?”), vividness (“How vivid is the correction?”), and imaginability (“While you were reading the correction, how easily could you picture the events taking place?”), all on 0 (not at all)–10 (very much) scales.

Results are summarized in Fig. 6. There was a large difference in story-ness between non-narrative and narrative corrections, with substantial differences also on vividness and imaginability dimensions. There was no difference between conditions on comprehensibility, and only a small difference on informativeness, which was to be expected given the narrative correction was designed to provide the same relevant corrective information plus the story “wrapper.” We concluded that our manipulation was implemented successfully.

### Experiment 2

**Claims and explanations.** On average, the non-narrative corrections had 101 words, with FRE = 40.83 and FKGL = 12.48; narrative corrections had 111.5 words, with FRE = 42.15 and FKGL = 12.1 (see Table 6). Affirmations had on average 87.5 words, with FRE = 52.9 and FKGL = 10.9 (see Table 7).

**Table 6:** Myths and their corresponding non-narrative and narrative corrections

Item number	Items	Non-narrative correction	Narrative correction
Myth-1	Gastritis and stomach ulcers are caused by excessive stress	There is now strong evidence that gastritis and stomach ulcers are caused by the bacterium Helicobacter pylori. Scientists Barry Marshall and Robin Warren are credited with the discovery of this association, which was viewed by the broader scientific community as novel. A Nobel Prize was awarded to Marshall and Warren because of this discovery. A consequence of this discovery is that antibiotics can be used to treat these conditions (WC = 69; FRE = 37.2; FKGL = 12.3)	Scientist Barry Marshall discovered that gastritis and stomach ulcers are caused by the bacterium Helicobacter pylori. At first, he was ridiculed by colleagues for his proposal. Frustrated, he intentionally drank a broth contaminated with the bacterium to prove that it caused disease. Soon after, Marshall developed gastritis as a result, and then successfully used antibiotics to treat himself. There is now strong evidence for the link, and the discovery earned Marshall and his colleague Robin Warren a Nobel Prize (WC = 79, ratio 1.14; FRE = 39.8; FKGL = 11.6)
Myth-2	Women talk more than men	Numerous studies have converged on the conclusion that females do not talk more than males. Based on studies recording regular speech fragments from volunteers, it has been estimated that both men and women say around 16,000 words a day. This type of research is often done by using a digital device that records 30 s of sound every 12.5 min over long periods of time. From this, the total number of words spoken per day can be extrapolated with satisfactory accuracy. Results indicate that there are outliers of both genders, meaning there are some people who speak much more and others who speak much less than the average (WC = 108; FRE = 47.0; FKGL = 12.0)	Females do not talk more than males. Professor James Pennebaker of the University of Texas was leisurely reading a magazine, when he encountered a claim that jolted his mind to action: that women are “chatterboxes” who speak three times as much as men. Dubious of the claim, he decided to test its validity. To do so, Pennebaker recorded the speech of hundreds of volunteers, who wore digital devices that recorded 30 s of sound every 12.5 min. After painstaking analysis, he found that both men and women say around 16,000 words a day, a finding that has been replicated in numerous other studies. Amusingly, the most talkative person in the study was a man, racking up 47,000 words a day! (WC = 120, ratio 1.11; FRE = 41.3; FKGL = 12.4)
Myth-3	Cracking your knuckles leads to arthritis	There is no correlation between cracking one’s knuckles and the development of arthritis, despite prevalent belief about the relationship. For example, one study demonstrated that frequent knuckle cracking did not lead to the development of arthritis in the hand, even in knuckles cracked up to 36,500 times over a time span of 50 years. The study, titled “Does knuckle cracking lead to arthritis of the fingers?”, was published in the scientific journal Arthritis and Rheumatism. Dr. Donald Unger, the sole author of the article, received the 2009 Ig Nobel Prize for the work. This is a prize which is awarded for research that makes you laugh, then think. (WC = 107; FRE = 43.5; FKGL = 12.4)	There is no correlation between cracking one’s knuckles and the development of arthritis—as was most convincingly shown by Dr. Donald Unger. When Unger was a child, his parents scolded him every time he cracked his knuckles, warning him, “you’re going to develop arthritis!” Curious about whether this was true, he began cracking his left-hand knuckles daily, while never cracking his right hand. After 50 years—cracking his left-hand knuckles about 36,500 times in the process—Unger had not developed arthritis in either hand. He published the finding in the scientific journal Arthritis and Rheumatism. For his work, Unger received the 2009 Ig Nobel Prize, awarded for research that makes you laugh, then think. (WC = 113, ratio 1.06; FRE = 45.4; FKGL = 11.5)
Myth-4	Delayed-onset muscle soreness is caused by buildup of lactic acid.	Lactic acid produced in muscles during strenuous exercise does not cause muscle soreness a day or two after exercise. Scientific evidence shows that strenuous exercise that a person is used to partaking in does not produce delayed-onset muscle soreness. Relatively easy exercise that a person is not used to, on the other hand, does produce muscle soreness. This occurs despite the fact that the relatively easier exercise often results in a lower level of lactic acid production, compared to the more strenuous but familiar exercise. Thus, delayed-onset muscle soreness is not the result of lactic acid buildup. Rather, the soreness is caused by micro-tears to muscle fibers, which are more likely to occur when engaging in new types of exercise. (WC = 120; FRE = 35.6; FKGL = 13.2)	Lactic acid produced in muscles during strenuous exercise does not cause muscle soreness. Sport scientist James Schwane, an avid runner, questioned the often-cited relationship between lactic acid and delayed-onset muscle soreness based on his own experience, and decided to test it. Schwane got participants to either run on a flat surface (which was strenuous, but involved movements the runners were used to), or downhill (which was easier, but less similar to runners’ usual movements). He discovered that running downhill produced less lactic acid but caused more soreness than running on a flat surface. This led him to conclude that delayed-onset muscle soreness is not linked to lactic acid. Rather, he concluded that the soreness is caused by micro-tears to muscle fibers, which are more likely to occur when engaging in new types of exercise (WC = 134, ratio 1.12; FRE = 42.1; FKGL = 12.9)

WC, Word Count; FRE, Flesch Reading Ease; FKGL, Flesch–Kincaid Grade Level

**Table 7:** Facts and their Corresponding Affirmations

Item	Claim	Affirmation
Fact A	Stomach acid can dissolve razor blades	A study in 1997 confirmed that our gastric juices can indeed dissolve razor blades, albeit slowly. This is possible due to simple chemistry: The lining of our stomach secretes hydrochloric acid, which dissolves many metals. Razor blades are made of steel, which is an alloy of iron, and are therefore readily dissolved by hydrochloric acid. The study concluded that, if you were to swallow a razor blade, the best time for surgery would be 15 h or so after ingestion. This is because by this time the blade will have become fragile and could be broken and removed in a piecemeal fashion (WC = 102; FRE = 53.4; FKGL = 10.8)
Fact B	It is not safe to talk on landline telephones when there is a thunderstorm.	It is, in fact, not safe to talk on a landline during a thunderstorm. The current in a lightning bolt can exceed 100,000 volts. Electrical wires are good transmitters of electricity, so when lightning strikes a house, it has the potential to move through the interconnected cables. Usually, the energy is simply absorbed into the ground, but it is possible for the current to travel through the landline’s cables and shock the person on the end of the phone line (WC = 80; FRE = 55.7; FKGL = 10.5)
Fact C	Dogs can smell cancer	Dogs perform better than state-of-the-art screening tests at detecting people with lung and breast cancer. This has been tested in a scientific setting. Cancer patients have traces of chemicals (like alkanes and benzene derivatives) in their breath, which dogs can detect in concentrations as small as a few parts per trillion. A study at the University of California showed that dogs correctly detected 99% of lung cancer breath samples and made a mistake with only 1% of samples from healthy controls (WC = 81; FRE = 48.4; FKGL = 11.5)
Fact D	We are taller in the morning than in the evening	We are taller in the mornings than the evenings due to the compression of our spine over the course of the day. When you are standing or sitting, there is pressure on the intervertebral discs, which causes water to be expelled. At night, when the spine is horizontal, water is reabsorbed by the disks. In 1935, De Puky measured 1216 participants between 5 and 90 years old, and found the average person was more than half an inch shorter in the evening than they were in the morning (WC = 87; FRE = 53.2; FKGL = 10.9)

WC, Word Count; FRE, Flesch Reading Ease; FKGL, Flesch–Kincaid Grade Level

**Test questionnaire.**

See Table 8.

**Table 8:** Claims and corresponding inference questions

Item	Claim	Inference question 1	Inference question 2	Inference question 3
Myth A	Gastritis and stomach ulcers are caused by excessive stress	Patients with stomach ulcers should avoid any type of stress	How effective do you think relaxation techniques are in preventing gastritis?	How likely is it that you would advise a friend or family member with stomach pains to reduce stress so they do not develop a stomach ulcer?
Myth B	Women talk more than men	At any given time, a woman is more likely to be speaking compared to a man	In general, jobs that require a lot of talking are a more natural fit for women	If you met a new male–female couple, how likely is it that the woman would talk more than the man?
Myth C	Cracking your knuckles leads to arthritis	People with a family history of arthritis should avoid cracking their knuckles	Children should be taught not to crack their knuckles in order to reduce the risk of arthritis in later life	How likely is it that you would advise a friend or family member with joint pains in their hands to avoid knuckle-cracking?
Myth D	Delayed-onset muscle soreness is caused by buildup of lactic acid	After strenuous exercise, a warm-down routine is essential because it breaks-down the lactic acid that contributes to delayed-onset muscle soreness	How effective do you think supplements that help break down lactic acid are in preventing exercise-induced muscle soreness?	How likely is it that you would advise a friend or family member with exercise-induced muscle soreness to avoid exercise activities that create lactic acid?
Fact A	Stomach acid can dissolve razor blades	Teaching teenagers that our stomach acid can dissolve razor blades would be an accurate and entertaining way to inform them about chemistry	How effective do you think stomach acid is at dissolving razor blades?	How likely is it that a razor blade would be totally intact after 48 h in stomach acid?
Fact B	It is not safe to talk on landline telephones when there is a thunderstorm	People should be discouraged from talking on landlines during thunderstorms to reduce their risk of being electrocuted	Even when inside, people should opt to use mobile phones instead of landlines during a thunderstorm	How likely is it that you would advise a friend or family member not to talk on a landline during a thunderstorm?
Fact C	Dogs can smell cancer	Sniffer dogs are a reliable and effective way to detect some cancers	Sniffer dogs trained to detect cancer should be utilized more in hospitals	To what extent would you trust the response of sniffer dog over a traditional screening test of lung cancer?
Fact D	We are taller in the morning than in the evening	If you are half an inch too short to go on a rollercoaster in the evening, how likely is it that you would be allowed to ride the following morning?	If you want to seem taller, you should measure yourself first thing in the morning	When doctors measure their patients, they should take into account the time of day

All inference questions are measured on 11-point Likert scales from 0 (strongly disagree) to 10 (strongly agree)

**Pilot study.** A different sample of 102 US-based MTurk workers (min. 5000 HITs completed with 98% + approval rate) was recruited to rate the non-narrative and narrative corrections of all real-world myths. One participant was excluded due to uniform responding (SD = 0), and one was excluded because they indicated we should not use their data due to lack of effort. This left N = 100 participants (M_age_ = 37.58 years; age range 21–65; 61 males, 39 females).

All myths were presented in randomized order. For each myth, participants read both corrections, also in randomized order. They were asked to rate each correction on informativeness (“How informative is the correction?”), comprehensibility (“How easy to understand is the correction?”), story-ness (“How story-like is the correction?”), and vividness (“How vivid is the correction?”), all on 0 (not at all)–10 (very much) scales. The imaginability dimension was omitted as the non-narrative correction featured no events that could have been pictured.

Results closely mirrored the findings from Experiment 1 Pilot and are summarized in Fig. 7. Again, there was a large difference in story-ness between non-narrative and narrative corrections, with a substantial difference also on vividness. There was no difference between conditions on comprehensibility, and only a small to-be-expected difference on informativeness. We again concluded that our manipulation was implemented successfully.

**Core analyses using preregistered exclusion criterion.** Core analyses were repeated excluding all participants with any initial myth-belief ratings of zero, as per the pre-registration. Results were equivalent to the analysis reported in the paper: In the two-way mixed ANOVA with factors condition and delay on myth-belief-change scores, the main effect of condition and the interaction were nonsignificant, F < 1. The planned contrasts of NN versus N conditions at either delay were also nonsignificant, F < 1. The ANOVA on inference scores yielded a significant main effect of condition, F(1,531) = 5.09, MSE = 2.38, η_p_^2^ = .009, p = .024, indicating lower scores in the narrative condition (F < 1 for the interaction). However, the core planned NN versus N contrast was nonsignificant in both the immediate test, F(1,531) = 3.71, η_p_^2^ = .007, p = .055, and the delayed test, F(1,531) = 1.60, η_p_^2^ = .003, p = .206.

### Experiment 3

**Claims and explanations. **On average, the non-narrative corrections had 112 words, with FRE = 45.55 and FKGL = 11.9; narrative corrections had 117.5 words, with FRE = 55.55 and FKGL = 10 (see Table 9). Affirmations had on average 86.5 words, with FRE = 37.1 and FKGL = 12.85 (see Table 10).

**Table 9:** Myths and their corresponding non-narrative and narrative corrections

Item number	Items	Non-narrative correction	Narrative correction
Myth-1	Humans are made to eat red meat; it should be part of every person’s diet	Recent research-based evidence published in a leading journal shows that eating red meat on a regular basis may shorten people’s lifespans. The findings of the study suggest that meat eaters might improve their health by making simple changes. One suggestion made is to substitute one serving of red meat (like bacon or steak) a day with another type of protein. Options include fish, chicken, legumes, low-fat dairy and whole grains. The results of the study suggest that rotating in other foods in place of red meat could lower the risk of mortality by 7 to 19% (WC = 96; FRE = 58.6; FKGL = 9.8)	“To me, there’s no finer pleasure than smelling bacon in the morning, or sinking my teeth into a perfectly cooked steak. You can imagine my panic when my daughter, who is a nurse, showed me research-based evidence that eating red meat frequently may shorten my lifespan! She asked, ‘Promise me you’ll make some changes? Just substitute one serving a day with another protein.’ With her help, I rotated in other foods like fish, chicken, legumes, low-fat dairy, and whole grains. She says that lowers my mortality risk by 7 to 19%. I still get to enjoy a sizzling steak on special occasions!” (WC = 102; 1.06 ratio; FRE = 66.8; FKGL = 7.5)
Myth-2	Children of homosexual parents have more mental health issues	A large body of research has examined the question of whether children of homosexual parents have poorer development outcomes. This research has looked at a wide range of social, emotional, health and academic outcomes. It has compared patterns of mental health and related outcomes in children with same-sex parents compared to children in more traditional households. This research shows that children or adolescents raised by same-sex parents fare equally as well as those raised by opposite-sex parents. An article published in the Journal of Marriage and Family in 2010 conducted a summary analysis of 33 individual studies on the topic. The results of the research review suggest that the strengths that are typically associated with mother–father families appear to the same degree in families with two same-sex parents (WC = 128; FRE = 32.5; FKGL = 14)	“People sometimes ask me what it’s like to have two mothers, rather than a mom and a dad. It seems to me like my family does the same things other, ‘normal’ families do. For a college project, I actually looked into the research and found that children or adolescents raised by same-sex parents fare equally as well as those raised by opposite-sex parents on a wide range of social, emotional, health and academic outcomes. One study, published in the Journal of Marriage and Family in 2010, analyzed the results of 33 individual studies to assess how the gender of parents affected children. The authors found that the strengths typically associated with mother-father families appear to the same degree in families with two same-sex parents. I certainly don’t feel any different than my peers!” (WC = 133; 1.04 ratio; FRE = 44.3; FKGL = 12.5)

WC, Word Count; FRE, Flesch Reading Ease; FKGL, Flesch–Kincaid Grade Level

**Table 10:** Facts and their Corresponding Affirmation

Item number	Items	Affirmation
Fact-1	Laughing regularly helps improve vascular function	It is well known that laughter reduces stress hormones and releases endorphins, yet strangely enough, it also has a positive impact on vascular function. A 2009 study found that people with heart disease were 40% less likely to laugh in a variety of situations compared to people without heart disease. A study in 2010 demonstrated the short-term benefits of laughter by showing participants either a 20-min clip of a comedy or a documentary. Laughter led to tissue dilation in the inner lining of blood vessels, which increased blood flow (WC = 90; FRE = 39.2; FKGL = 13.3)
Fact-2	US citizens are the most generous people in the world	US citizens are consistently rated the most generous people in the world. Be it volunteering their time, donating money to charity, or helping out a stranger in need, the World Giving Index reports that 58% of Americans regularly partake in an act of generosity. That is more people per capita than any other country. In 2018 alone, US citizens donated a staggering $292 billion dollars to charity. More than half of individuals reported that financial constraints were stopping them from donating even more! (WC = 83; FRE = 35.0; FKGL = 12.4)

WC, Word Count; FRE, Flesch Reading Ease; FKGL, Flesch–Kincaid Grade Level

**Test questionnaire.**

See Table 11.

**Table 11:** Myths and facts, and corresponding inference questions

Item number	Items	Inference question 1	Inference question 2	Inference question 3
Myth-1	Humans are made to eat red meat; it should be part of every person’s diet	Meals served to children at schools should include at least one serving of red meat every day	To maintain a healthy diet, people should regularly consume red meat	Diets and health care plans that do not include red meat are unsustainable for humans
Myth-2	Children of homosexual parents have more mental health issues	School counselors should be trained to look for characteristics of anxiety and depression in children of homosexual couples	Children whose parents are homosexual are at an increased risk of experiencing mental health issues	Homosexual couples considering adoption should consider the impact of their homosexuality on the child’s mental health
Fact-1	Laughing regularly helps improve vascular function	Laughing workshops should be recommended for people with cardiovascular diseases	The American Heart Association should run an advertisement campaign promoting laughter as a preventative measure for heart disease	People should be advised to watch comedies as a way to improve their heart health
Fact-2	US citizens are the most generous people in the world	Americans should be regarded as generous people	Americans can be proud of their generosity	Charities seeking funds would be well advised to target Americans as potential donors

**Pilot study.** A separate sample of N = 100 US-based MTurk workers (min. 5000 HITs completed with 98% + approval rate; M_age_ = 36.43 years; age range 20–70; 57 males, 43 females) was recruited to rate the non-narrative and narrative corrections of both controversial real-world myths.

Both myths were presented in randomized order. For each myth, participants read both corrections, also in randomized order. They were asked to rate each correction on informativeness (“How informative is the correction?”), comprehensibility (“How easy to understand is the correction?”), story-ness (“How story-like is the correction?”), and vividness (“How vivid is the correction?”), all on 0 (not at all)–10 (very much) scales.

Results closely mirrored the findings from the Experiment 2 Pilot and are summarized in Fig. 8. Again, there was a large difference in story-ness between non-narrative and narrative corrections, with a substantial difference also on vividness. There was no difference between conditions on comprehensibility, and only a moderate difference on informativeness (with the non-narrative correction being rated somewhat more informative, which was expected given the narrative correction provided more arbitrary, conversational information). We again concluded that our manipulation was implemented successfully.

## Footnotes

1 Prolific (https://www.prolific.co/) is a recruitment platform known for high-quality data (e.g., Peer et al. 2017).

2 Different criteria for immediate and delayed test were set after initial peer review as part of the pre-registration, which occurred before data collection.

3 Although it can be assumed that corrections can reduce claim belief even in participants with relatively low levels of initial belief (e.g., a reduction from 2 to 1 or 1 to 0), naturally no reduction is possible from zero. In the pre-registration, the criterion was specified as “any initial-belief ratings of zero”; it was stated that, should final sample size *n* drop below 352 in either delay condition (the min. sample size suggested by power analysis), we would resample (352 − *n*) × 1.25 participants in the immediate condition (to again account for zero-belief and other exclusions), and/or (352 − *n*) × 1.5 participants in the delayed condition (to account for zero-belief and other exclusions, as well as dropout due to delay) prior to analysis. We also stated that these values might be adjusted based on the actual rejection and dropout rates we observe. However, applying this strict criterion (even applying it only to myth beliefs, which was the intention) would have resulted in 350 + exclusions; we thus decided to relax this criterion. As this is a deviation from pre-registration, we report the results of the core analyses applying the stricter, preregistered criterion in “Appendix”. Results were statistically equivalent to those reported in “Results” section below.

4 We recruited participants who responded with “conservative” to the Prolific prescreener “Where would you place yourself along the political spectrum?” (conservative, moderate, liberal, other).

5 There is evidence for a link between political conservatism and meat consumption (Gallup 2018; Hodson and Earle 2018) as well as negative attitudes toward homosexuality (Haslam and Levy 2006; McLeod et al. 1999; Terrizzi et al. 2010).

6 We note that the non-narrative corrections were rated as somewhat more informative; this was not surprising given that the narrative corrections contained some conversational elements. This makes our test more conservative: results illustrating that narrative corrections are more effective than non-narrative ones would imply that the story factor can even overcome a slight informativeness deficit.

7 We acknowledge that a person can have low belief in a claim they would like to believe based on their worldview, and thus it is possible that there would still be a narrative advantage in the lower belief range. However, in Experiment 3 we aimed to create corrections that challenged participants’ worldview-consistent beliefs, which will only be true if initial belief in that misinformation is at least at a moderate level. In the initial, peer-reviewed manuscript, we thus specified the exclusion criterion as “any initial myth-belief rating < 2, or both initial ratings < 3”; in the pre-registration (after peer review but before data collection for Experiment 3), we specified that we would apply this criterion unless it would lead to more than 25% of data being rejected, at which point we would relax the criterion to “any initial myth-belief rating < 1, or both initial ratings < 2”. The stricter criterion would have led to 256 exclusions (approx. 27% of data overall); hence, we relaxed the criterion as per the preregistered plan.

8 An exploratory test using a directed alternative hypothesis H_1_ in terms of a narrative benefit (i.e., N < NN rather than N ≠ NN) yielded BF_01_ = 52.87, which can be interpreted as very strong evidence against a narrative benefit.
